# Molecular Signatures of Natural Killer Cells in CMV-Associated Anterior Uveitis, A New Type of CMV-Induced Disease in Immunocompetent Individuals

**DOI:** 10.3390/ijms22073623

**Published:** 2021-03-31

**Authors:** Nobuyo Yawata, Mariko Shirane, Kaing Woon, Xinru Lim, Hidenori Tanaka, Yoh-Ichi Kawano, Makoto Yawata, Soon-Phaik Chee, Jay Siak, Koh-Hei Sonoda

**Affiliations:** 1 Department of Ocular Pathology and Imaging Science, Kyushu University, Fukuoka 812-8582, Japan; 2 Singapore Eye Research Institute, Singapore 168751, Singapore; woonkaing@gmail.com (K.W.); limxr@imcb.a-star.edu.sg (X.L.); chee.soon.phaik@singhealth.com.sg (S.-P.C.); jay.siak.j.k@singhealth.com.sg (J.S.); 3 Ophthalmology and Visual Sciences Academic Clinical Program, Duke-NUS Medical School, Singapore 169857, Singapore; 4 Department of Ophthalmology, Kyushu University, Fukuoka 812-8582, Japan; marikoshirane22@gmail.com (M.S.); sonodak@med.kyushu-u.ac.jp (K.-H.S.); 5 HLA Foundation Laboratory, Kyoto 600-8813, Japan; h-tanaka@hla.or.jp; 6 Department of Ophthalmology, Fukuoka Dental College, Fukuoka 814-0193, Japan; ykawano@college.fdcnet.ac.jp; 7 Singapore Institute for Clinical Sciences (SICS), Agency for Science, Technology and Research, A*STAR, Singapore 117609, Singapore; paeym@nus.edu.sg; 8 Department of Pediatrics, Yong Loo Lin School of Medicine, National University of Singapore, Singapore 119228, Singapore; 9 National University Health System, Singapore 119228, Singapore; 10 Immunology Programme, Life Sciences Institute, National University of Singapore, Singapore 117456, Singapore; 11 NUSMED Immunology Translational Research Programme, National University of Singapore, Singapore 117456, Singapore; 12 International Research Center for Medical Sciences, Kumamoto University, Kumamoto 860-8555, Japan; 13 Department of Ophthalmology, Yong Loo Lin School of Medicine, National University of Singapore, Singapore 119228, Singapore; 14 Ocular Inflammation and Immunology Department, Singapore National Eye Centre, Singapore 168751, Singapore

**Keywords:** cytomegalovirus, cytomegalovirus-associated anterior uveitis, killer cell immunoglobulin-like receptors, HLA class I, natural killer cells, CD57, KLRG1, NKG2C

## Abstract

Cytomegalovirus (CMV) causes clinical issues primarily in immune-suppressed conditions. CMV-associated anterior uveitis (CMV-AU) is a notable new disease entity manifesting recurrent ocular inflammation in immunocompetent individuals. As patient demographics indicated contributions from genetic background and immunosenescence as possible underlying pathological mechanisms, we analyzed the immunogenetics of the cohort in conjunction with cell phenotypes to identify molecular signatures of CMV-AU. Among the immune cell types, natural killer (NK) cells are main responders against CMV. Therefore, we first characterized variants of polymorphic genes that encode differences in CMV-related human NK cell responses (Killer cell Immunoglobulin-like Receptors (*KIR*) and *HLA class I*) in 122 CMV-AU patients. The cases were then stratified according to their genetic features and NK cells were analyzed for human CMV-related markers (CD57, KLRG1, NKG2C) by flow cytometry. *KIR3DL1* and *HLA class I* combinations encoding strong receptor–ligand interactions were present at substantially higher frequencies in CMV-AU. In these cases, NK cell profiling revealed expansion of the subset co-expressing CD57 and KLRG1, and together with KIR3DL1 and the CMV-recognizing NKG2C receptor. The findings imply that a mechanism of CMV-AU pathogenesis likely involves CMV-responding NK cells co-expressing CD57/KLRG1/NKG2C that develop on a genetic background of KIR3DL1/HLA-B allotypes encoding strong receptor–ligand interactions.

## 1. Introduction

Human cytomegalovirus (HCMV) causes latent infection in 50–90% of global populations and is prevalent especially in Asian populations [1]. Various mechanisms have been proposed to explain how CMV evades immune responses to maintain latent infection, resulting in recurrent, asymptomatic, subclinical reactivation of CMV [2]. The clinical manifestations of latent CMV infection are thought to occur primarily in severely immunocompromised individuals, such as in those with Acquired Immunodeficiency Syndrome and patients who are undergoing transplantation [3]. In the field of ophthalmology, CMV retinitis has been well-documented in these conditions [4]. However, the recent, newly established entity of CMV-anterior uveitis (CMV-AU) is noteworthy in that recurrent, intraocular inflammation is induced by CMV in individuals without apparent immunodeficiency or immune suppression [5,6]. Furthermore, newly developed, high-sensitivity, PCR-based detection methods have revealed that CMV is likely the most frequent cause of virus-induced anterior uveitis in Chinese and Japanese populations [7,8]. In terms of treatments for CMV-AU, topical combination therapy with the anti-viral drug ganciclovir and corticosteroids is effective; however, the inflammation recurs if treatment is discontinued [9]. This observation implies that the pathogenesis of CMV-AU likely involves dysregulation of the host immunity that controls CMV reactivation.

Studies on the immunological mechanisms of CMV infection have focused primarily on either severely immunocompromised cases or healthy human controls. The reasons are yet unclear as to why some individuals without apparent immunosuppression develop recurrent, CMV-induced ocular inflammation. CMV-AU is prevalent in Asian populations and occurs primarily in individuals who are middle-aged and older [7,10]. These clinical observations suggest that genetic factors combined with the consequences of immunological senescence are critical factors in the pathological mechanism in this disease. Based on this hypothesis, we designed this study to investigate genetic factors and immunological senescence in natural killer (NK) cells, which have been identified as major responders in the immune response against CMV [11,12].

The functions of NK cells differ substantially among human individuals [13], where cellular responses are regulated via the interactions between Killer cell Immunoglobulin-like Receptors (KIRs) and their cognate ligands, HLA class I. In contrast to CD8^+^T cells, which are stimulated by upregulated expression of HLA class I, NK cells are activated by downregulation of HLA class I, namely the ‘missing-self response’, which is an immune response unique to NK cells. This has relevance in infection or malignant transformation where HLA class I molecules will often become downregulated [14]. Furthermore, the genetic polymorphisms in KIRs and HLA class I have been demonstrated to differentiate the strength of missing-self responses; the concept of ‘NK cell education’ or ‘licensing’ [15,16,17,18]. Therefore, inherited *KIR* and *HLA class I* polymorphisms are directly responsible for differences in cytotoxic and pro-inflammatory NK cell responses [13,19].

The two main modes of immunological escape by CMV involve either (1) downregulation of HLA-A and HLA-B in infected cells, or (2) loading of peptides derived from a CMV-encoded protein, UL40, onto HLA-E [20,21,22]. The former mechanism results in loss of cytotoxic T cell responses; however, in this situation, the host can still respond against CMV-infected cells through NK cell responses mediated by the missing-self mechanism [14]. Of relevance to this study is the extensive variability in the genes that confer individuality to NK cell licensing and the anti-CMV response: HLA class I and the KIR (KIR3DL1 in particular in this study). KIR3DL1 recognizes only the HLA-B allotypes with a Bw4 motif (approximately 1/3 of the HLA-B allotypes); therefore, the induction of NK cell missing-self responses via this mechanism depends on the *HLA-B* alleles of the individual [17].

In the latter mechanism of immune evasion, the responses of NK cell subpopulations expressing the HLA-E specific inhibitory receptor NKG2A are suppressed [20,21,22]. In this situation, the presence of NK cells expressing the HLA-E binding-activating NKG2C receptor is of importance in CMV infection, where NKG2C binds to the HLA-E/UL40 complex to initiate anti-virus NK cell responses. The NKG2C-positive NK cell subpopulation is apparently expanded permanently in CMV-seropositive individuals; thus, the presence of this expanded subset in peripheral blood has been considered a hallmark of past CMV infection [23]. Such alterations to otherwise stable NK cell repertoire structures have been reported for several types of virus infections, such as those by human immunodeficiency virus, and the changes imply substantial involvement of NK cells in the anti-virus immune response [24]. One goal of this study was to identify this immunological signature in CMV-AU cases.

We hypothesized that there may be some features in *KIR*-*HLA* genetics as a background condition, and secondly that the recurrent inflammation characteristic of CMV-AU is triggered by dysregulated NK cell functions. To investigate these questions, we first stratified the cases according to genetic factors with the most potential to alter human NK cell function, and then studied NK cell differentiation markers in CMV-AU blood specimens. We then characterized the genetic polymorphisms of *HLA class I* and *KIR* in 122 CMV-AU cases and integrated the data with NK cell phenotype analyses. Our findings suggest a role for NK cells co-expressing CD57, KLRG1, and NKG2C that have developed on a genetic background of KIR3DL1/HLA-B allotypes encoding strong receptor–ligand interactions, in CMV-AU pathogenesis.

## 2. Results

### 2.1. Polymorphisms of KIRs and Their Ligands Associated with CMV-AU

**Figure 1:**
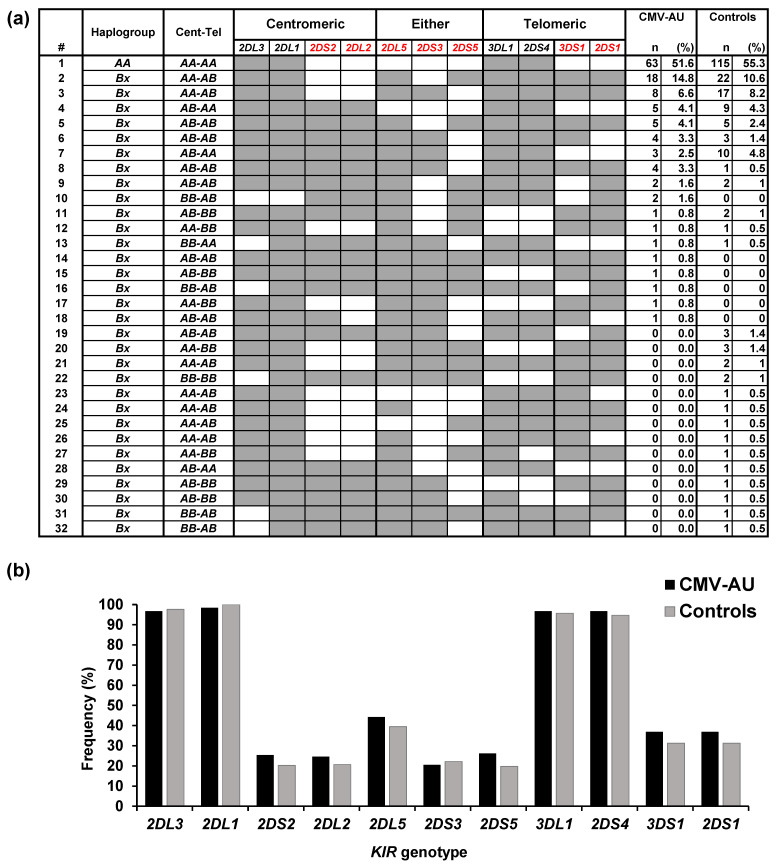
*KIR* genotypes in CMV-associated anterior uveitis (CMV-AU) patients and healthy controls. *KIR* genotypes (**a**) and gene frequencies (**b**) were determined in 122 individuals with CMV-AU. As a control, *KIR* genotypes in 208 healthy individuals of the same ethnicity (Chinese Singaporean) are shown from a previous study [25]. CMV-seropositivity was 87% based on a previous study in this population [26]. (**a**) Shaded boxes indicate presence of *KIR* genes. The centromeric/telomeric position of *KIR* loci indicated in columns 4 and 6 are their physical location within the KIR complex on chromosome 19 [27,28]. *KIR2DL5*, *KIR2DS3*, and *KIR2DS5* genes can locate on either centromeric/telomeric sides of the *KIR* complex and are thus indicated in this table as such (column 5). *KIR B* haplotype-specific *KIR* genes are shown in red. *KIR* framework genes and pseudogenes are excluded. (**b**) Black and gray boxes indicate each *KIR* genotype frequency in CMV-AU and healthy controls, respectively.

*KIR* and *HLA class I* genotypes were characterized in 122 CMV-AU cases, and the profiles were compared with those of 208 healthy individuals of the same ethnicity (Chinese Singaporean) [25] (Figure 1a). Eighteen *KIR* genotypes were identified in the CMV-AU cases, six of which were present only in CMV-AU. In contrast, 14 of the 26 *KIR* genotypes present in healthy controls were not found in CMV-AU. No apparent association of individual *KIR* genes with CMV-AU was detected in this study (Figure 1b).

In the human genome, the 16 *KIR* genes cluster in one area of chromosome 19 where the genome structure is highly variable, resulting in many haplotypes carrying different combinations of *KIR* gene loci [27]. In the context of anti-CMV NK cell responses, KIR2DL1, KIR2DL2, KIR2DL3, and KIR3DL1 are of importance due to their roles in programming missing-self NK responses, the concept of NK cell education, or licensing [15,16,17]. Whereas many *KIR* haplotypes carry various combinations of these four *KIR* genes (in addition to others), one particular haplotype—the group *A KIR* haplotype with a fixed set of seven *KIR* genes containing *KIR2DL1*, *KIR2DL3*, and *KIR3DL1*—was of particular interest in our study, as this *A* haplotype is present at a high frequency in East Asian populations [29,30]. All other combinations of *KIR* genes are collectively termed group *B KIR* haplotypes [18]. However, in this study, no apparent association between group *A KIR* haplotypes (51.6% homozygotes in CMV-AU, 55.3% in the healthy controls) and CMV-AU was detected (Figure 1a,b).

We next applied the classification of *KIR* haplotype blocks that have proven useful in allogeneic bone marrow transplantation as surrogate predictors of NK cell alloreactivity [31]. In this system, fixed sets of *KIR* genes in linkage disequilibrium on the haplotypes are classified into centromeric and telomeric *KIR* genomic motifs [31]. This classification enables a rough estimation of the activation potential of NK cells in the individual based on the tendency of *KIR B* haplotypes to carry a higher frequency of activating *KIR* genes.

The *KIR* genotypes in the CMV-AU cohort were assigned *KIR* haplotype block groupings based on whether the genotypes contained genome segments derived from *A* or *B* haplotypes in the centromeric (Cen) or telomeric (Tel) sides of the *KIR* gene complex on chromosome 19 (Table 1, details in Methods section). This classification revealed a significantly higher frequency of the Cen-*AB* + Tel-*AB KIR* motif groups (heterozygous for groups *A* and *B* haplotype blocks on both the centromeric and telomeric *KIR* genes) in the CMV-AU cases compared with the healthy controls.

KIR function is dependent on the presence/absence of the cognate HLA class I as its ligand; therefore, we characterized the HLA class I allotype groups (*HLA-C* groups 1 and 2 and *HLA-B* Bw4-positive *HLA-B* alleles; details in the Methods section) that were assumed to serve as KIR ligands in the CMV-AU cases. The frequencies of these KIR ligand groups in the CMV-AU group did not differ significantly compared with those in the healthy control group (Table 2). However, when the HLA-B allotypes were compared, allotypes with isoleucine at position 80 (80Ile) were over 3 times more frequent than the alternative threonine (80Thr) at this position in the CMV-AU group (80Ile/80Thr ratio: CMV-AU 3.1 vs. control 0.90). This is of importance from the perspective of NK cell reactivity because engagement of HLA-B molecules with 80Ile results in significantly stronger signaling through the cognate KIR3DL1 receptor [19,32].

Associations between CMV-AU and *KIR* polymorphisms were also identified. KIR allotypes differ in cell surface expression levels, inhibitory capacity, and licensing [13,17,19,33]. To enable better assessment of the potential for *KIR* polymorphisms in driving the pathogenesis of anterior uveitis, we compared KIR allotype frequencies between CMV-AU and CMV-seropositive healthy control cohorts (Table 3). Here, we identified seven *KIR3DL1* alleles in these two cohorts and found that 86% of CMV-AU and CMV-seropositive controls had high expression alleles of *KIR3DL1* (*KIR3DL1H*) (Table 4). This was significant because KIR3DL1H allotypes have been demonstrated to confer strong NK cell licensing in combination with HLA-B Bw4 80Ile [19,32]. Overall, the genetic analyses indicated a substantial presence of strong inhibitory KIR-HLA interactions in the CMV-AU cohort.

### 2.2. Identification of Four NK Cell Subsets Distinguished by Differential Expression of CD57 and KLRG1: Expansion of CD57^+^KLRG1^−^ and CD57^+^KLRG1^+^ NK Cell Populations in CMV-Seropositive Individuals

We investigated the known CMV-related markers in the NK cell populations in individuals carrying the genetic background of *HLA-B Bw4 80Ile* and *KIR3DL1H*. CD57, NKG2C, and specific KIRs were markers of interest as previous studies have demonstrated that CMV infection induces expansion of NK cells expressing them. In particular, Beziat et al. reported that NK cells expressing inhibitory KIRs that recognize self-ligands (self-iKIR) are often selectively expanded during CMV infection [34]. CD57 is also an established marker for terminally differentiated human NK cells and has been used to assess immunological senescence. In contrast, KLRG1, which is considered a maturation marker in rodent NK cells, is not regarded to be a maturation marker in human NK cells [35].

**Figure 2:**
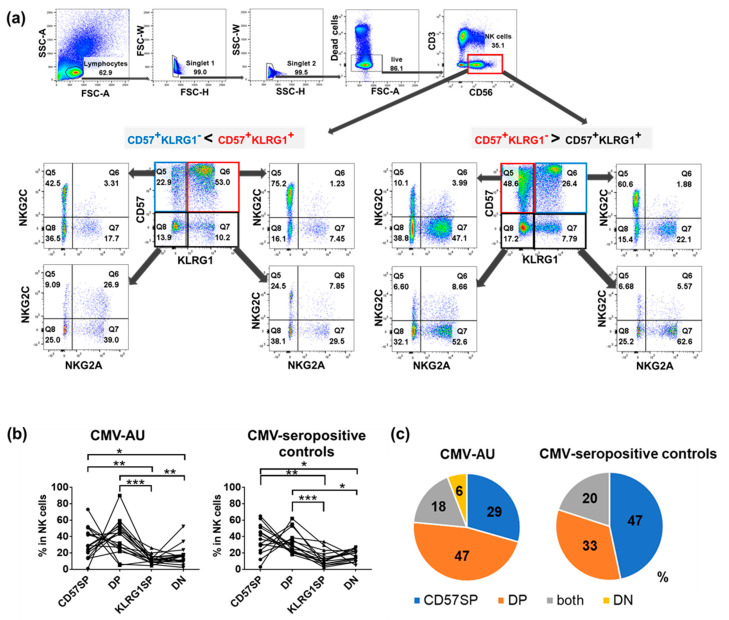
Expansion of the CD57^+^KLRG1^−^ or the CD57^+^KLRG1^+^ subsets among the four natural killer (NK) cell subsets distinguished by the differential expression of CD57 and KLRG1. (**a**) Identification of four NK cell subsets as determined by differential expression of CD57 and KLRG1. Representative profiles of two CMV-seropositive healthy individuals with expansion of either the CD57^+^KLRG1^+^ or CD57^+^KLRG1^−^NK subset. Notably, NKG2A and NKG2C are expressed at different levels on each subset. (**b**) Frequencies of the four NK subsets are shown for each individual. ANOVA: CMV-AU, *p* = 0.0025; CMV-seropositive controls, *p* = 0.002. * *p* < 0.05, ** *p* < 0.01, *** *p* < 0.001. (**c**) The proportions of NK cell subsets most expanded in the CMV-AU and CMV-seropositive healthy individuals. CMV-AU: *n* = 17, CMV-seropositive controls: *n* = 15.

High-dimensional flow cytometric analysis of NK cells in CMV-AU and CMV-seropositive healthy individuals revealed the existence of four distinct subsets distinguished by various combinations of CD57 and KLRG1 expression as follows: CD57 single positive (CD57 SP), CD57/KLRG1 double positive (DP), KLRG1 single positive (KLRG1 SP), and CD57/KLRG1 double negative (DN) subsets (Figure 2a,b). Importantly, these four subsets expressed different levels of NKG2A and NKG2C, implying that they are functionally distinct. The NK cell profiles in CMV-AU cases tended to differ from those of the CMV-seropositive healthy individuals, with the CD57/KLRG1 DP subset present at the highest frequency in the CMV-AU cohort (47%, 7 of 15 individuals) and the CD57SP subset present at the highest frequency in the CMV-seropositive healthy individuals (47%, 8 of 17 individuals) (Figure 2c).

### 2.3. CD57/KLRG1 DP Subset Expansion in CMV-AU Individuals with HLA-B Bw4 80Ile and KIR3DL1H

**Figure 3:**
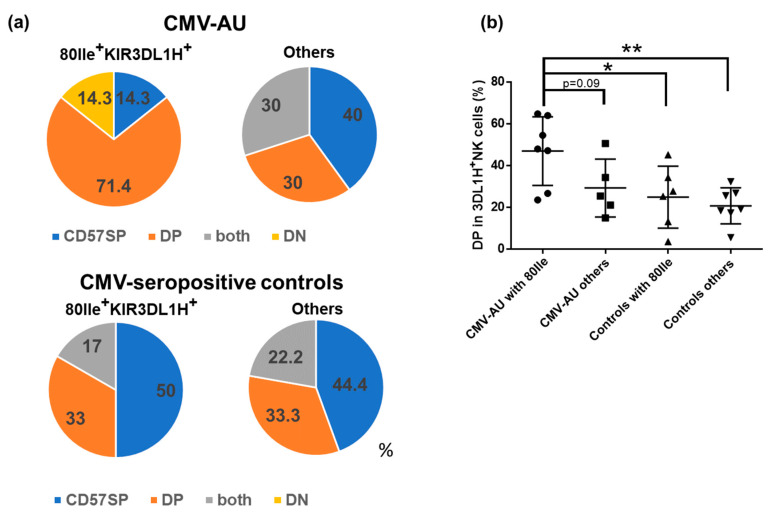
Expansion of CD57/KLRG1 DP NK cells in CMV-AU patients carrying HLA-B Bw4 80Ile and KIR3DL1H. (**a**) The proportion of expanded NK cell subsets among CMV-AU patients and CMV-seropositive healthy individuals in the presence or absence of HLA-B Bw4 80Ile and KIR3DL1H (CMV-AU, *n* = 17; CMV-seropositive controls, *n* = 15). (**b**) Distributions of KIR3DL1H^+^NK cells among the CD57/KLRG1 subsets. CD57/KLRG1 DP subset frequencies among KIR3DL1H^+^NK cells were significantly elevated in CMV-AU patients carrying HLA-B Bw4 80Ile as compared to CMV-seropositive controls (CMV-AU, *n* = 12; CMV-seropositive controls, *n* = 13; ANOVA *p* = 0.0097). Data represent the mean+ standard deviation. * *p* < 0.05, ** *p* < 0.01.

We next investigated the potential influence of the presence of self-ligands for inhibitory KIRs on the NK cell profiles in the CMV-AU cohort. Having identified that *KIR3DL1H* alleles combine with strong HLA-B Bw4 allotypes as a feature of the genetic background in CMV-AU, we investigated the CD57/KLRG1 NK cell phenotypes in association with CMV-AU cases with this background. As shown in Figure 3a, we observed a significant expansion of the CD57/KLRG1 DP population in 71.4% of patients carrying HLA-B Bw4 80Ile and KIR3DL1H. In contrast, the CD57SP population was the most frequent (44–50%) in the CMV-seropositive controls, regardless of whether or not the individuals carried HLA-B Bw4 80Ile and KIR3DL1H. Furthermore, the ratio of CD57/KLRG1 DP NK cell population was significantly higher among the KIR3DL1-expressing NK cells in CMV-AU individuals carrying HLA-B Bw4 80Ile and KIR3DL1H compared to the CMV-seropositive controls (Figure 3b). These results imply an interesting profile of NK cells in the CMV-AU cohort where the CD57/KLRG1 DP NK cell population has apparently expanded in patients carrying a genetic background of *HLA-B Bw4 80Ile* and *KIR3DL1H* alleles.

### 2.4. Differential Expression of NKG2C and iKIRs in CD57SP and DP NK Subsets

**Figure 4:**
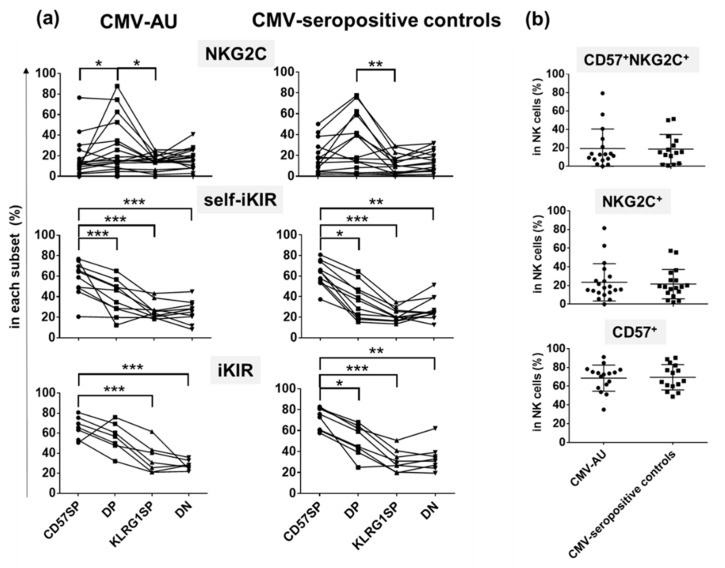
The four NK cell subsets defined by differential expression of CD57 and KLRG1 express disparate levels of NKG2C and iKIR. (**a**) Proportion of cells expressing NKG2C and iKIR on the four NK cell subsets. (CMV-AU, *n* = 15; CMV seropositive controls, *n* = 17. ANOVA: NKG2C, *p* = 0.047; self-iKIR, *p* = 0.0001; iKIR, *p* = 0.0006. Linear trend test: self-iKIR & iKIR, *p* < 0.0001). * *p* < 0.05, ** *p* < 0.01, *** *p* < 0.001. (**b**) Proportion of CD57^+^NKG2C^+^, NKG2C^+^, CD57^+^NK cells in CMV-AU and CMV-seropositive healthy individuals (CD57^+^NKG2C^+^, CD57^+^: CMV-AU, *n* = 17; CMV-seropositive controls, *n* = 15; NKG2C^+^: CMV-AU, *n* = 20; CMV-seropositive controls, *n* = 18). Data represent the mean ± standard deviation.

We then sought to understand the implications of the NK cell profiles in our cohorts. We found significant elevation of NKG2C expression on the CD57/KLRG1 DP subset both in CMV-AU patients and CMV-seropositive controls (Figure 4a). As NKG2C directly recognizes a CMV virus protein and induces expansion of NKG2C-expressing NK cells, expansion of the CD57/KLRG1 DP subset in CMV-AU is likely due to the strong activation induced by CMV-infected cells [21,22,36,37]. In contrast, no difference was observed in the frequencies of CD57^+^NKG2C^+^, CD57^+^, and NKG2C^+^NK cells between CMV-AU and CMV-seropositive controls (Figure 4b).

We also identified distinct expression patterns of self-iKIRs on CD57/KLRG1 subsets. Among the CD57/KLRG1 subsets, the CD57SP subset had the highest frequency of cells expressing self-iKIRs followed by the CD57/KLRG1 DP subset, KLRG1 SP subset, and the CD57/KLRG1 DN subset in both CMV-seropositive healthy controls and CMV-AU (Figure 4a).

## 3. Discussion

To the best of our knowledge, this is the first study investigating immunological factors in CMV-AU. The individuals tended to carry KIR3DL1 and HLA-B allotypes encoding strong inhibitory receptor–ligand interactions, and our findings show substantial presence of NK cell populations in these cases co-expressing CD57 and KLRG1 receptors in conjunction with the receptor NKG2C that binds the CMV UL40 virus protein.

CMV is known to downregulate HLA-B expression on infected cells. This leads to the induction of NK cell missing-self responses in the KIR3DL1-expressing NK cell subsets, especially in donors carrying both the HLA-B Bw4 80Ile and KIR3DL1H allotypes. These allotype combinations are one of those that have been demonstrated to confer strong NK cell licensing [19,32].

CMV also modulates expression of multiple HLA class I moieties as an escape mechanism from both T cell and NK cell immunity. In this context, T cell escape is achieved by inducing downregulation of classical HLA class I [20]; however, this has the potential to trigger NK cell missing-self responses depending on the HLA allotypes of the host. As a possible means to effectively escape NK cell responses in this situation, the virus induces upregulation of the non-classical HLA class I molecule, HLA-E. Previous studies have shown that this process is mediated by enhancing the loading of HLA-E molecules with peptides derived from the virus UL40 protein [22]. The HLA-E:UL40 peptide complex is then recognized by the inhibitory NK cell receptor, NKG2A, to inhibit NK cell responses.

HLA-E is also recognized by the activating NK cell receptor, NKG2C. In this study, NKG2C was expressed mainly on the expanded CD57/KLRG1 DP subset. Thus, we hypothesize that NKG2C expression on the CD57/KLRG1 DP subset has facilitated recognition of CMV-infected cells.

The previous reports that have described inhibitory KIR co-expression in the CD57^+^NKG2C^+^NK cell populations expanded in CMV-seropositive individuals prompted us to investigate composite KIR and HLA genetics in our CMV-AU cases [34,38,39]. In this study, we identified KIR3DL1H allotypes in donors carrying strong HLA-B allotypes (80Ile), which led to our hypothesis for strong KIR–ligand interactions playing a role in the pathogenesis of CMV-AU, possibly as a result of enhanced activation induced by missing-self responses.

It is noteworthy that the high expression allotypes of KIR3DL1 and HLA-B allotypes with 80Ile polymorphisms are both present at higher frequencies in East Asian as compared to Caucasian populations [7,13,40,41,42], corresponding to the global distribution of CMV-AU.

We also identified significantly higher expression of iKIRs on CD57SP NK cells compared to CD57/KLRG1 DP subsets. During NK cell development, immature NK cells are known to express HLA-specific receptors at different stages, where NKG2A is expressed exclusively in stage 4 and iKIR expression follows thereafter in stage 5 [43]. This implies that the CD57SP NK subset expanded in our CMV-seropositive controls displays a more mature phenotype compared to the CD57/KLRG1 DP subset expanded in CMV-AU patients. We speculate that the potential difference in maturation status of the expanded NK subset in CMV-AU patients compared with asymptomatic CMV-seropositive controls could be an additional factor contributing to the pathogenesis of CMV-AU.

Polymorphisms of *KIR*s and their ligands have been associated with various clinical issues in CMV infection. Patients with symptomatic, primary CMV infection tend to carry allotypes of HLA-B that serve as a weaker ligand (80Thr allotypes) for KIR3DL1 [44]. These patients also tended to be homozygous for the group *A KIR* haplotypes (that carry KIR3DL1), implicating NK cell inhibition through KIR3DL1 interactions as a mode of pathogenesis. Compared with healthy controls, our analyses showed a contrasting genetic background where CMV-AU cases tended to carry heterozygous combinations of group *A* and *B KIR* haplotypes, rather than being homozygous for group *A KIR* haplotypes (high frequencies of the Cen *A*/*B* + Tel *A*/*B* haplotype block combinations). These *KIR* genotypes encode a balanced mix of inhibitory and activating KIR in these cases. Thus, we hypothesize that CMV-AU may have a different mechanism of pathogenesis compared to symptomatic primary CMV infection.

Studies have indicated that the presence of more activating *KIR* genes (encoded primarily in the *KIR B* haplotypes) is protective against CMV reactivation in hematopoietic stem cell transplantation, implying potent roles of activating KIRs and NK cells in controlling CMV reactivation [45,46,47]. Given the prevalence of *KIR B* haplotypes (encoding more activating KIRs) and the expansion of the CD57/KLRG1 DP NK cell subset (co-expressing NKG2C and KIR3DL1H) in CMV-AU patients with the HLA-B Bw4 80Ile allotypes, our current hypothesis is that the higher gene dose of activating KIR in CMV-AU cases results in dysregulated activation of the CMV-specific NKG2C-expressing NK cell subsets.

Neither the CMV-AU patients nor the CMV-seropositive healthy individuals displayed extraocular symptoms that have been hitherto associated with CMV infection in general, such as colitis and hepatitis. Recent studies have also inferred an association with CMV-seropositivity and increased prevalence of cardiovascular diseases and increased morbidity/mortality in elderly populations [48]. In our cohort, the CMV-AU patients did not seem to manifest CMV-associated symptoms other than in the eye; however, it will be of interest to screen for CMV-induced changes (some of which may be subclinical), and especially in the context of aging and tissue senescence, and the risks as a consequence of these conditions. A second point of importance as a feature of CMV-AU is that the inflammation is apparently limited to the intraocular space—an immune-privileged site. This observation begs the question whether other immune-privileged tissues similarly develop inflammation in CMV-AU cases. This remains an unanswered question in this pilot study.

Although many studies have employed the use of animal models to investigate CMV-induced disease, we intentionally limited our approach to the analyses of clinical specimens in this study. It is difficult to study clinical isolates of human CMV in other species due to host specificity, and also due to virus instability caused by the high rate of virus mutations that occur in in vitro cultures. The AD169 laboratory strain of human CMV has been often used in immunological studies; however, this strain was unsuitable for our study as it lacks the genes related to evasion from NK cell responses. Therefore, it will be important to test our hypothesis on the immunological modes of CMV-AU pathogenesis in a suitable model, once a viable platform is developed.

## 4. Materials and Methods

### 4.1. Human Subjects

One hundred and twenty-two Chinese Singaporean patients diagnosed as CMV-AU by PCR analysis of aqueous humor samples were recruited at the Singapore National Eye Centre from 2014 to 2017. Whole blood samples were collected from CMV-AU patients and genomic DNA was extracted using the Nucleon BACC Genomic DNA Extraction kit (GE Healthcare Life Sciences, MA, USA) according to the manufacturer’s instructions. Samples collected from 43 CMV-seropositive Chinese Singaporean healthy donors without ocular inflammation served as healthy controls in *KIR3DL1* allele analyses. Peripheral blood mononuclear cells (PBMCs) were isolated by Ficoll gradient separation from 20 CMV-AU (mean age 62 (43–81) years old) and 18 CMV-seropositive healthy controls (mean age 58 (37–76) years old) and compared for NKG2C expression on NK cells. Seventeen CMV-AU and 15 CMV-seropositive healthy controls were compared for differences in NK cell repertoires.

This study was conducted in accordance with the Declaration of Helsinki and was approved by the SingHealth Institutional Review Board. Written informed consent was received from all participants.

### 4.2. KIR Genotyping, KIR3DL1 Allele Typing and KIR Ligand Typing

PCR-based sequence-specific primer typing (PCR-SSP) for sixteen *KIR* genes was performed in 122 CMV-AU individuals with primer sets as described by Vilches et al. [49]. PCR was conducted using 0.2 μL of Platinum Taq polymerase (Thermo Fisher Scientific, Carlsbad, CA, USA) in 12 μL PCR reactions at 3.8 mM of MgCl_2_ and 150 ng genomic DNA as previously described [50]. The PCR conditions were initial denaturation for 2 min at 95 °C, then 10 cycles of 10 sec at 94 °C and 40 sec at 65 °C and 20 cycles of 20 sec at 94 °C, 20 sec at 61 °C and 30 sec at 72 °C, final extension of 7 min at 72 °C. All reactions were conducted using thermal cycler PE9700 (Applied Biosystems, Foster city, CA, USA). KIR ligand types were determined by typing *HLA-B* and *C* loci using a commercial system (Capture HLATM protocol and Assign MPS; Conexio Genomics, Perth, Australia) in 119 CMV-AU. Typing for *HLA-B* failed in three individuals. KIR3DL1 ligands (*HLA-B* alleles with the Bw4 motif) were classified into *80Ile* and *80Thr* groups by amino acids at position 80. *HLA-C* alleles were classified into group 2 (*C2*) and group 1 (*C1*) as cognate ligands for KIR2DL1 and KIR2DL3, respectively [18]. Centromeric *A/B* and telomeric *A/B KIR* motifs were assigned according to the presence or absence of *B KIR* haplotype-specific *KIR* genes in centromeric/telomeric *KIR* haplotype motifs according to Cooley et al. [31]. *KIR3DL1* alleles were determined by next-generation sequencing (NGS) using a commercial system (Illumina MiSeq combined with the *KIR* IGS system; Scisco Genetics, Seattle, WA, USA) [51].

### 4.3. Antibodies and Flow Cytometry

The following monoclonal antibodies were used for flow cytometric analysis: anti-CD56 (B159), anti-CD3 (SK7), anti-KIR2DL2/3/2DS2 (GL183), anti-CD57 (NK-1) (BD Biosciences, San Jose, CA, USA), anti-KIR3DL1 (DX9), anti-NKG2A (REA110), anti-KLRG1 (REA261), anti-KIR2DS4 (JJC1.6), anti-NKG2C (REA205) (Miltenyi Biotech, Bergisch Gladbach, Germany), and anti-KIR2DL1 (Cl143211) (R&D Systems, Minneapolis, MN, USA). Dead cells were excluded using LIVE/DEAD Fixable Dead Cell Staining kits (Thermo Fisher Scientific) according to the manufacturer’s instructions. After staining of PBMCs with LIVE/DEAD Fixable Dead Cell Stain for 10 min at room temperature, PBMCs were washed and stained with the antibody mixture for 30 min on ice. PBMCs were fixed with BD Cytofix (BD Biosciences) and analyzed by 11-parameter flow cytometry using a LSR Fortessa cell analyzer (BD Biosciences). Flow cytometry data were analyzed using FlowJo software (Tree Star, Ashland, OR, USA). Expanded NK subsets were defined by a more than 1.5-fold increase in frequency compared against other subsets. The frequencies of inhibitory KIRs in NK subsets were calculated in group *A* homozygotes to exclude activating KIRs recognized by anti-KIR2DL2/3/2DS2 mAbs.

### 4.4. Statistical Analysis

Data were statistically analyzed using Prism software (GraphPad Software; San Diego, CA, USA). *KIR* gene and *KIR* ligand frequencies were compared with healthy individuals of the same ethnicity (Chinese Singaporean, *n* = 208, unknown CMV-serostatus) in a previous study using Fisher’s exact test [25]. The CMV-seropositivity of the healthy controls was assumed to be more than 87%, based on a previous study [26]. Mann–Whitney tests were used for comparisons between two groups. Repeated-measures one-way ANOVA with Dunnett’s multiple comparison test was used for multiple comparisons between NK cell subsets. Linear trend tests were conducted for the analysis of receptor expression on NK subsets. *p*-values < 0.05 were considered statistically significant.

## 5. Conclusions

A mechanism of overly active CMV-specific NK cells is proposed in CMV-AU pathogenesis where NK cells with CMV-reactive phenotypes (co-expression of CD57, KLRG1, NKG2C) are expanded upon a genetic background encoding strong KIR-HLA interactions.

## Figures and Tables

**Table 1:** Comparison of *B*-content score-based *KIR* haplotype motifs between CMV-AU and healthy controls.

Haplotype Group	*B* Content	Motif		CMV-AU		Controls	
	Score	Cen	Tel	n	(%)	n	(%)
*AA*	0	*AA*	*AA*	63	51.6	115	55.3
*Bx*	1	*AA*	*AB*	26	21.3	45	21.6
		*AB*	*AA*	8	6.6	20	9.6
	2	*AA*	*BB*	2	1.6	5	2.4
		*AB*	*AB*	17	13.9	14	6.7 *
		*BB*	*AA*	1	0.8	1	0.5
	3	*AB*	*BB*	2	1.6	4	1.9
		*BB*	*AB*	3	2.5	2	1
	4	*BB*	*BB*	0	0	2	1

CMV-AU, *n* = 122; Healthy controls, *n* = 208 [25].Assignment of *A*/*B KIR* haplotype motifs and calculation of *B* content scores followed the method described by Cooley et al. [31]. * *p* = 0.049, Odds Ratio 2.24, 95% confidence interval (CI) 1.06–4.73.

**Table 2:** Frequency of *KIR* ligands in CMV-AU and healthy controls.

	**CMV-AU**		**Controls**		***P***		
	**n**	**%**	**n**	**%**			
*C1^+^*	118	99.2	206	99	n.s.		
*C2^+^*	27	22.7	45	21.6	n.s.		
	**CMV-AU**		**Controls**		***P***	**OR**	**95% CI**
	**n**	**%**	**n**	**%**			
***HLA-B Bw4^+^***	69	59.5	122	58.7	n.s.		
***80Ile^+^***	54	46.6	63	30.3	0.0058	1.94	1.23–3.1
***80Thr_+_***	17	14.7	70	33.7	0.0007	0.38	0.21–0.67
**ratio of *80Ile/80Thr***		3.1		0.9			

CMV-AU, HLA-C *n* = 119, HLA-B *n* = 116; healthy controls, *n* = 208 [25]. n.s.; no significance. OR; Odds Ratio.

**Table 3:** *KIR3DL1* allele-level genotypes in CMV-AU patients and CMV-seropositive healthy controls.

*KIR3DL1*	*KIR3DL1*	Phenotypes	CMV-AU		CMV-IgG ^+^ Controls	
(1st Allele)	(2nd Allele)		n	%	n	%
3DL1*001	3DL1*01502	HH	6	4.9	2	4.7
3DL1*01502	3DL1*01502	HH	21	17.2	15	34.9 *
3DL1*01502	3DL1*020	HH	2	1.6	0	0
3DL1*01502	3DL1*029	HH	2	1.6	0	0
3DL1*020	3DL1*001	HH	0	0	1	2.3
3DL1*001	3DL1*00501	HL	3	2.5	2	4.7
3DL1*00501	3DL1*01502	HL	23	18.9	5	11.6
3DL1*00501	3DL1*02901	HL	2	1.6	0	0
3DL1*00701	3DL1*01502	HL	11	9	3	7
3DL1*00701	3DL1*020	HL	1	0.8	0	0
3DL1*01502	3DL1*008	HL	0	0	1	2.3
3DL1*01502	*N*	H	29	23.8	7	16.3
3DL1*020	*N*	H	2	1.6	1	2.3
3DL1*029	*N*	H	3	2.5	0	0
3DL1*00501	3DL1*00501	LL	2	1.6	0	0
3DL1*00501	3DL1*00701	LL	1	0.8	0	0
3DL1*00701	3DL1*00701	LL	1	0.8	2	4.7
3DL1*00501	*N*	L	7	5.7	3	7
3DL1*00701	*N*	L	1	0.8	0	0
3DL1*00501	3DL1*038	L ?	1	0.8	0	0
*N*	*N*		4	3.3	1	2.3

CMV-AU, *n* = 122; CMV-IgG^+^ (seropositive) healthy controls, *n* = 43. H: high, L: low, N: absent, ?: undetermined. * *p* = 0.02, Odds Ratio: 0.4, 95% CI: 0.17–0.85.

**Table 4:** Frequency of individuals carrying *KIR3DL1H* alleles in the CMV-AU and CMV-seropositive control cohorts.

	CMV-AU		CMV-IgG ^+^ Healthy Controls	
	n	%	n	%
KIR3DL1H^+^	105	86.1	37	86

CMV-AU, *n* = 122; CMV-IgG^+^ (seropositive) healthy controls, *n* = 43.

## Data Availability

The data presented in this study are available in this article.

## Abbreviations

CMV	Cytomegalovirus
CMV-AU	CMV-associated anterior uveitis
NK cells	natural killer cells
KIRs	Killer cell Immunoglobulin-like Receptors
PBMC	Peripheral blood mononuclear cells
PCR-SSP	PCR-based sequence-specific primer typing
